# Serum HBV pregenomic RNA is correlated with Th1/Th2 immunity in treatment‐naïve chronic hepatitis B patients

**DOI:** 10.1002/jmv.25612

**Published:** 2019-11-21

**Authors:** Yurong Gu, Lubiao Chen, Yifan Lian, Lin Gu, Yaqiong Chen, Yanhua Bi, Zexuan Huang, Yanlin Huang, Bo Hu, Yuehua Huang

**Affiliations:** ^1^ Department of Infectious Diseases The Third Affiliated Hospital of Sun Yat‐sen University Guangzhou China; ^2^ Guangdong Provincial Key Laboratory of Liver Disease Research The Third Affiliated Hospital of Sun Yat‐sen University Guangzhou China; ^3^ Department of Laboratory Medicine The Third Affiliated Hospital of Sun Yat‐sen University Guangzhou China

**Keywords:** chronic, hepatitis B, immunity, RNA

## Abstract

**Background and Aim:**

Hepatitis B virus (HBV) load and antigens are related to the innate and adaptive immunity of chronic hepatitis B (CHB) patients. As a new HBV biomarker, the role of pregenomic RNA (pgRNA) in host immunity is not known. This study aimed to identify the relationship between serum HBV pgRNA and host immunity in CHB patients.

**Methods:**

Two hundred twenty‐five treatment‐naïve CHB patients were enrolled. Serum cytokines were measured by cytokine antibody array (Luminex multiplex platform). Th1 (T‐helper cell, Th) and Th2 cells were tested by flow cytometry. Serum HBV pgRNA was detected by a reverse transcription‐polymerase chain reaction.

**Results:**

Serum HBV pgRNA was significantly different among patients in different disease phases and significantly associated with both HBV antigens and antibodies. Serum HBV pgRNA was positively correlated with the HBsAg level (*P* < .001) and the presence of HBeAg (*P* < .001). Patients with higher HBcAb levels showed lower serum HBV pgRNA levels (*P* = .003). Notably, HBsAb positivity was associated with higher levels of serum HBV pgRNA in HBeAg(−) patients (*P* = .049). Serum HBV pgRNA was positively associated with ALT level, Th2 cell frequency, and related cytokine sCD30 (*P* < .001, *P* < .001, and *P* = .003, respectively), but negatively associated with Th1‐related cytokine interleukin (IL)‐12P70 and cytotoxic lymphocytes (CTLs) (*P* = .017 and *P* < .001, respectively).

**Conclusion:**

Our study confirmed the relationship between serum HBV pgRNA and host immunity. The results demonstrated that serum HBV pgRNA is positively correlated with Th2 immunity but negatively correlated with Th1 immunity, indicating that it might have a relationship with HBV antigen conversion and CTL immunodeficiency in CHB patients.

## Highlights

- Serum HBV pgRNA had a relationship with host immunity.
- Serum HBV pgRNA was positively correlated with Th2 cells and cytokines.
- Serum HBV pgRNA was negatively correlated with CTLs and Th1 cytokine.

## 1 INTRODUCTION

Chronic hepatitis B virus (HBV) infection still affects more than 240 million people worldwide and induces the development of liver cirrhosis, liver failure, and hepatocellular carcinoma.^1^ HBV is noncytopathic to hepatocytes, and HBV‐related liver damage is caused by chronic immune inflammation. During the course of liver disease, adaptive immunity is crucial for the pathogenesis of hepatic inflammation, especially T cells, which play important roles in the antiviral response.^2^, ^3^, ^4^ T‐cell activity largely depends on the differentiation of naïve T cells into effector T cells or T‐helper cell subsets by releasing cytokines.^5^ In addition to T cells, studies on chronic hepatitis B (CHB) patients in different disease phases identified a genetic signature of rigorous B‐cell activation in patients in the chronic active phase.^6^ In HBV infection, the importance of B cells is limited to not only their ability to produce neutralizing antibodies but also to act as potent antigen‐presenting cells, particularly for helper T cells,^7^ and have been shown to play a regulatory role in that context. Interleukin‐10 (IL‐10)‐producing B cells are enriched in CHB patients, particularly during hepatic flares, and have been shown to modulate not only inflammatory events but also HBV‐specific T‐cell responses.^8^

HBV virus DNA load and antigens, such as HBsAg and HBeAg, are thought to be related to the innate and adaptive immunity of CHB patients. For example, as the HBV DNA load increases, the activation and function of immune cells are suppressed. Sustained exposure to high quantities of viral antigens is thought to contribute to defects in T‐cell function by interacting with and modulating T cells directly or through the induction of immunosuppressive cytokines and the inflammatory liver environment.^9^, ^10^, ^11^, ^12^

Intrahepatic covalently closed circular DNA (cccDNA) is the transcription template of all HBV transcripts, and HBV produces messenger RNAs (mRNAs), 2.4 and 2.1 kb surface mRNAs and 0.7 kb X mRNA, and the 3.5 kb pregenomic RNA (pgRNA) intracellularly.^13^, ^14^, ^15^, ^16^ Gene sequences of HBV RNA were found to be comparable in serum and liver tissue, indicating that serum HBV RNA originates from the infected hepatocytes.^17^ However, the mechanism underlying the release of HBV RNA into serum from infected hepatocytes remains unclear.^18^ The available study results suggested that serum HBV RNA species might vary depending on the detection method, CHB stage or antiviral treatment.^17^, ^19^, ^20^, ^21^, ^22^, ^23^, ^24^, ^25^, ^26^ However, serum HBV RNA is probably a mixture of intact, spliced, and polyA‐free pgRNA, and the detected serum HBV RNA sequences are predominantly derived from pgRNA.^18^ Transcription from cccDNA to pgRNA is an important step in HBV replication, and pgRNA is regarded as the template for the reverse transcription and synthesis of the HBV genome.^27^, ^28^, ^29^ Serum HBV pgRNA can be detected in the sera of CHB patients,^20^, ^21^, ^22^ although its levels are significantly lower than HBV DNA levels.^27^ Serum HBV pgRNA is regarded as an important biomarker that could reflect the persistence of HBV infection, HBV replication and rebound,^19^, ^30^ intrahepatic cccDNA transcriptional activity in the liver tissues of CHB patients,^30^, ^31^ and is also regarded as an early predictor of antiviral therapy,^20^, ^21^, ^22^, ^26^ emergence of HBV drug resistance, or safe withdrawal of nucleoside analog (NUC) therapy.^32^

Since serum HBV pgRNA has recently been regarded as an important new biomarker reflecting HBV replication and HBV‐related immune‐mediated inflammatory liver injury is induced by components of the virus, it is important to determine whether serum HBV pgRNA also has a relationship with host immunity, similar to HBsAg or HBV DNA, in CHB patients. However, little is known about this relationship. In this study, HBV antigens and antibodies, serum cytokine profiles, Th1/Th2 cells, and serum HBV pgRNA were examined in a relatively large cohort of treatment‐naïve CHB patients to identify the relationship between serum HBV pgRNA and host immunity.

## 2 SUBJECTS AND METHODS

### 2.1 Patients

Two hundred twenty‐five treatment‐naïve CHB were recruited from the hepatitis clinic at the Third Affiliated Hospital of Sun Yat‐sen University. Patients who were previously receiving antiviral treatment (interferon or nucleoside analogs), were coinfected with human immunodeficiency virus, hepatitis C virus, or hepatitis D virus, had an autoimmune disease or fatty liver disease, were receiving immunosuppressive therapy, or had cirrhosis or malignancies were excluded. Written informed consent was obtained from all patients before their inclusion in the study. The study conforms to the Declaration of Helsinki and was approved by the Institute Review Board of Sun Yat‐sen University. Demographic and laboratory information is listed in Table 1. Healthy controls were simultaneously recruited from The Physical Examination Center.

**Table 1:** Clinical‐virological characteristics of CHB patients included in the study

Characteristics	HBeAg(−) (n = 125)	HBeAg(+) (n = 100)	*P* value
Age (mean ± SD), y	33.6 ± 8.2	27.5 ± 5.2	***.000***
Sex			.08
Male, n (%)	94 (75.2)	64(64)	
Female, n (%)	31 (24.8)	36 (36)	
BMI (mean ± SD)	21.85 ± 2.58	20.88 ± 2.70	***.014***
FibroScan (mean ± SD), kPa	5.38 ± 2.02	6.28 ± 3.81	.06
ALT (mean ± SD), U/L	50.36 ± 119.63	82.90 ± 124.77	***.04***
TBIL (mean ± SD), µmol/L	15.41 ± 18.70	18.43 ± 28.56	.349
qHBsAg (mean ± SD), log_10_ IU/mL	2.9 ± 0.83	4.21 ± 0.64	***.000***
HBV DNA (mean ± SD), log_10_ IU/mL	3.35 ± 1.63	7.42 ± 1.54	***.000***
HBcAb, COI	0.01 ± 0.011	0.013 ± 0.018	.259
HBsAb			.680
Positive	14	13	
Negative	111	87	
HBV genotype			***.000***
B, n (%)	59 (47.2)	59 (59)	
C, n (%)	24 (19.2)	25 (25)	
O, n (%)	5 (4)	8(8)	
NA, n (%)	37 (29.6)	8 (8)	
HBV mutation			***.000***
C, (n%)	30 (24)	28 (28)	
PreC, (n%)	32 (25.6)	14 (14)	
BCP, (n%)	22 (17.6)	18 (18)	
NA, (n%)	70 (56)	6 (6)	

*Note*: HBV genotype O: besides B or C, including genotype D, B + D, B + C, or C + D.

Abbreviations: BCP, basal core promoter; BMI, body mass index; CHB, chronic hepatitis B; HBV, hepatitis B virus; NA, not detected.

### 2.2 Clinical and serological parameters

HBsAg, anti‐HBsAg, HBeAg, and HBeAb were tested using commercial kits (Abbott Laboratory, North Chicago, IL). qHBsAg was measured using Elecsys HBsAg II Quant reagent kits (Roche Diagnostics, Indianapolis, IN). HBcAb levels were semiquantified with a chemiluminescence immunoassay (Roche Diagnostics). Serum HBV DNA levels were measured by Roche COBAS AmpliPrep/COBAS TaqMan HBV Test v2.0 (range from 20 to 1.7E+08 IU/mL; Roche Molecular Diagnostics, Branchburg, NJ). HBV genotypes and C/PreC/basal core promoter (BCP) mutations were determined by direct sequencing. Healthy controls had no history of liver or other diseases, did not have HBV infection, and had normal liver functions.

### 2.3 Quantification of serum HBV pgRNA

Serum HBV pgRNA levels were tested as described by Wang et al^19^ and van Campenhout et al^33^ with modifications. We used the EasyPure Viral RNA Kit (TransGen Biotech, Beijing, China) to isolate serum HBV RNA and treated it with DNase I (Thermo Fisher Scientific, Waltham, MA). A RevertAid First Strand cDNA Synthesis Kit (Thermo Fisher Scientific) with either random or HBV‐specific RT primers was used to reverse transcribe the isolated HBV pgRNA. The sequence of the HBV‐specific RT primer was 5′‐ATTCTCAGACCGTAGCACACGACACCGAGATTGAGATCTTCTGCGAC‐3′ in which the random sequence ATTCTCAGACCGTAGCACACGACAC was anchored at the 5′ end of the HBV‐specific sequence CGAGATTGAGATCTTCTGCGAC (nt 2436‐2415). Serum HBV DNA was detected by quantitative real‐time polymerase chain reaction (qPCR) in a LightCycler 480 II Real‐time PCR Detection System (Roche, Mannheim, Germany). The primers and probe used to detect HBV pgRNA were as follows: F (nt 2295‐2312): 5′‐AYAGACCATCAAATGCCC‐3′, R (anchored sequence): 5′‐ATTCTCAGACCGTAGCACACGACAC‐3′ and probe: 5′‐FAM‐CTTATCAACACTTCCGGARACTACTGTTGTTAGAC‐BHQ1‐3′. The standards for HBV pgRNA were obtained by PCR using each primer pair from HepG2.215 cDNA. The PCR products were ligated into the pEASY‐Blunt Cloning Vector (TransGen Biotech). The qPCR mixture (30 µL) contained 15 µL of 2X mix (LightCycler 480 Probes Master; Roche), 1 µL of forward primer (10 µM), 1 µL of reverse primer (10 µM), 1 µL of TaqMan probe (10 µM), 3 µL of cDNA template, and 9 µL of double‐distilled water. The reaction mixture was denatured at 95°C for 5 minutes, followed by 40 cycles at 95°C for 20 seconds and 60°C for 40 seconds. To ensure that no HBV DNA was measured, the RNA samples were analyzed by qPCR without the reverse transcription enzyme at the same time. The limit of detection is 25 copies/mL.

### 2.4 Cytokine profiles tested by protein array

Twenty‐five cytokines and chemokines in serum were tested using a Luminex multiplex cytokine assay kit (eBioscience, San Diego, CA) according to the manufacturer's instructions, including interferon‐γ (IFN‐γ), tumor necrosis factor‐α (TNF‐α), IL‐1β, IL‐2, IL‐4, IL‐6, IL‐7, IL‐8, IL‐12P40, IL‐12P70, IL‐15, IL‐17a, IL‐17C, IL‐22, IL‐23, IL‐28α, IL‐29, CC chemokine ligand MDC‐CCL22, MIP‐1α‐CCL, and MIP‐3α‐CCL2. The CXC‐chemokine ligand MIG‐CXCL9, IP‐10, I‐TAC, TNF receptor superfamily member sCD30, and cytokine receptor superfamily member gp130 were measured in a protein array to demonstrate innate and adaptive immune responses and chemokine responses to HBV infection. For example, the activation of Th1 cells is indicated by IFN‐γ, IL‐2, IL‐7, IL‐12, and IL‐15; Th2 cells are indicated by IL‐4, IL‐10, and sCD30; Th17 cells are indicated by IL‐1β, IL‐17, IL‐21, and IL‐23; Th22 cells are indicated by IL‐22; and IL‐6, IL‐29, and IL‐28α cells reflect innate immune responses.

### 2.5 Th1/Th2 cells

Peripheral blood monouclear cells (PBMCs) were stimulated with Leukocyte Activation Cocktail (eBioscience) at 37°C for 4 hours before intracellular staining using the PharMingen staining protocol. The cells were stained with PE‐CF594‐CD3, APC‐CD4, and V450‐CD8 monoclonal antibody (BD Biosciences) for 30 minutes at 4°C and then fixed and permeabilized using Cytofix/Cytoperm fixation/permeabilization solution (eBioscience) according to the manufacturer's instructions. The cells were stained with fluorescein isothiocyanate‐IFN‐γ, PE‐IL‐2, PE‐IL‐4, and PE‐CY7‐TNF‐α (eBioscience) for 45 minutes on ice and then washed. Corresponding isotype‐matched controls were purchased from BD Biosciences and eBioscience. Data were acquired on a Gallios instrument (Beckman Coulter, Brea, CA) and analyzed with FlowJo software (Ashland, OR).

### 2.6 Statistical analysis

The average levels of serum HBV pgRNA are described as median values (quartile spacing) and expressed in the logarithm10 form. Samples with undetectable serum HBV pgRNA were assigned a value of 0 log_10_ copies/mL for the data analysis. Nonparametric test analyses were used. We compared patient groups using the Mann‐Whitney *U* test or the Wilcoxon signed‐rank test (two samples) and the Kruskal‐Wallis test (*k* samples) for continuous variables and the *χ* ^2^ test for categorical variables. Associations between variables were tested using Spearman correlation or other nonparametric equivalents when appropriate. Linear regression analysis was performed to determine factors associated with serum HBV pgRNA levels. All other statistical tests were performed using SPSS version 23. Statistical significance was set to 0.05.

## 3 RESULTS

### 3.1 Distribution of serum HBV pgRNA levels in current cross‐sectional treatment‐naïve CHB patients

Defined by serum ALT levels and HBeAg status, patients were divided into four groups according to 2017 EASL guidelines.^34^ In group I (n = 56, HBeAg(+) and normal ALT [≤40 U/L]), 51 out of 56 (91.1%) patients were serum HBV pgRNA‐positive. In group II (n = 44, HBeAg(+) and elevated ALT [>40 U/L]), 42 out of 44 patients (95.5%) were serum HBV pgRNA‐positive. In group III (n = 93, HBeAg(−) and normal ALT [≤40 U/L]), 49 out of 93 patients (52.7%) were serum HBV pgRNA‐positive. In group IV (n = 32, HBeAg(−) and elevated ALT [>40 U/L]), 20 out of 32 patients (62.5%) were serum HBV pgRNA‐positive. The average serum HBV pgRNA levels of the four groups were 6.05 (4.66, 6.58), 6.67 (5.96, 7.91), 0.74 (0, 2.46), and 1.96 (0, 3.62) log_10_ copies/mL, respectively. Serum HBV pgRNA levels were significantly different among the patient groups and were higher in group II than in the other three groups (*P* = .041). The serum HBV PgRNA levels of group I were significantly higher than those of groups III and IV (*P* < .001). However, the serum HBV pgRNA levels in groups III and IV were comparable (*P* = .316) (Figure S1A).

The distribution of serum HBV pgRNA levels was also examined among the immune tolerant (IT), immune active (IA), inactive carriers (IC), and gray zone (GZ) phases categorized according to the American Association for the Study of Liver Disease guidelines.^1^ The average serum HBV pgRNA levels of patients at the four phases were 6.05 (5.72, 6.36), 5.93 (2.41, 6.89), 0.00 (0.00, 1.81) and 1.24 (0.00, 3.13) log_10_ copies/mL, respectively. The average levels of serum HBV pgRNA in patients in both IT and IA groups were higher than those in GZ and IC groups (*P* < .001). However, there were no statistically significant differences in serum HBV pgRNA levels between IT and IA groups (*P* > .05) or between GZ and IC groups (*P* > .05) (Figure S1B).

### 3.2 Association with HBV antigens and antibodies

**Figure 1:**
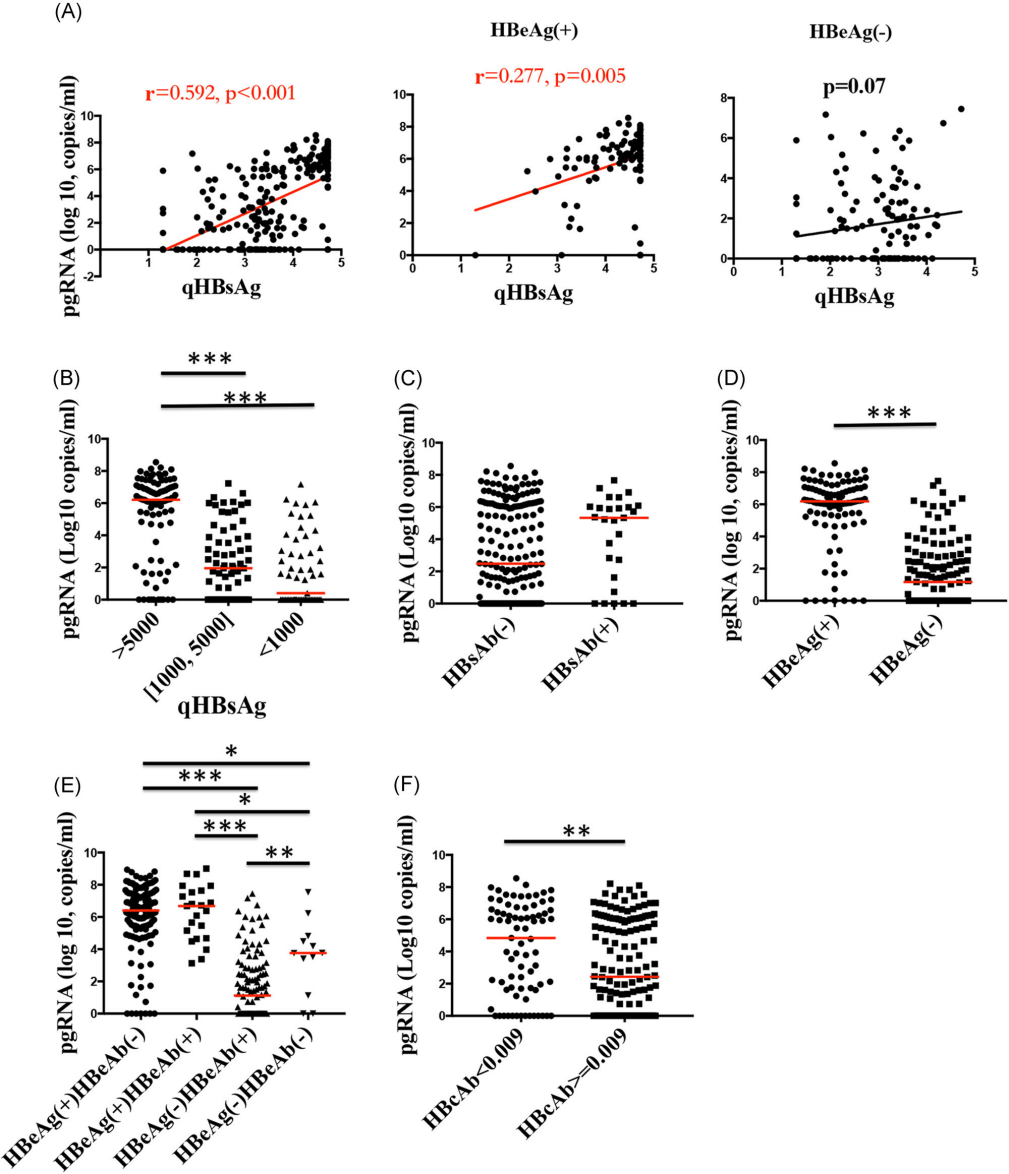
Association between HBV antigens and antibodies. A, The correlation between serum HBV pgRNA and qHBsAg in all CHB patients and HBeAg(+) patients was significant, while no obvious correlation was observed between them in HBeAg(−) patients. B‐F, Serum HBV pgRNA levels were different among patients with different qHBsAg levels, HBsAb, HBeAg, HBeAb, and HBcAb statuses. HBV, hepatitis B virus; pgRNA, pregenomic RNA. *P < .05, **P < .01, ***P < .001

A significant correlation was observed between serum HBV pgRNA and HBsAg levels in all patients by Spearman correlation analysis (*r* = .592; *P* < .001). Among HBeAg(+) patients, the Spearman correlation coefficient of serum HBV pgRNA and HBsAg was .277 (*P* = .005). However, no significant correlation was observed between serum HBV pgRNA and HBsAg levels (*P* = .07) in HBeAg(−) patients (Figure 1A). We further studied the relationship of serum HBV pgRNA levels with HBsAg by stratifying patients into three groups according to their qHBsAg titers: >5000 IU/mL (n = 97, 43.1%), 1000‐5000 IU/mL (n = 69, 30.7%), and <1000 IU/mL (n = 59, 26.2%). Patients with qHBsAg titers >5000 IU/mL had significantly higher serum HBV pgRNA levels than patients with qHBsAg titers of 1000‐5000 IU/mL (6.21 [4.74, 7.01] vs 1.95 [0, 3.92] log_10_ copies/mL; *P* < .001) or <1000 IU/mL (6.21 [4.74, 7.01] vs 0.41 [0, 3.04] log_10_ copies/mL; *P* < .001). There was no difference in serum HBV pgRNA levels between the groups with qHBsAg titers between 1000‐5000 and <1000 IU/mL (*P* = .232) (Figure 1B). Although compared with CHB patients with negative HBsAb, patients with both positive HBsAg and HBsAb did not have a higher level of serum HBV pgRNA (5.33 [1.62, 6.96] vs 2.48 [0.00, 6.16] log_10_ copies/mL; *P* = .213) (Figure 1C), there was a tendency for the serum HBV pgRNA levels of the above two groups to differ in HBeAg(−) patients (2.78 [0.00, 5.22] vs 0.74 [0.00, 2.41] log_10_ copies/mL; *P* = .057]. In our later enlarged cohort study, in which the number of patients was increased to 323, the difference in serum HBV pgRNA levels between HBeAg(−) patients with HBsAb(+) and HBsAb(−) became significant (2.83 [0.00, 5.37] vs 1.10 [0.00, 2.70] log_10_ copies/mL; *P* = .049).

Moreover, serum HBV pgRNA levels were significantly higher in HBeAg(+) patients than in HBeAg(−) patients (6.19 [5.40, 7.01] vs 1.17 [0.00, 2.80] log_10_ copies/mL; *P* < .001) (Figure 1D). We then compared patients with different HBeAg and HBeAb statuses and found no difference in serum HBV pgRNA levels between HBeAg(+) patients regardless of the HBeAb status (*P* = .808). Serum HBV pgRNA levels were obviously lower in HBeAg(−) patients with HBeAb(+) than in those with an HBeAb(−) status (3.76 [1.70, 4.74] vs 1.12 [0.00, 2.78] log_10_ copies/mL; *P* = .005) in the enlarged cohort (Figure 1E).

A negative correlation was observed between the serum HBV pgRNA and HBcAb values in all patients as analyzed by Spearman correlation (*r* = −.266; *P* < .001). Patients with higher HBcAb values (n = 148; HBcAb ≥0.009 COI) had significantly lower serum HBV pgRNA levels than those with lower HBcAb values (n = 77; HBcAb <0.009 COI) (2.46 [0, 6.03] vs 5.13 [1.66, 6.66] log_10_ copies/mL; *P* = .003) (Figure 1F). Notably, in HBeAg(+) patients, the correlation between serum HBV pgRNA and HBcAb was found to be significant by Spearman correlation (*r* = −.248; *P* = .013). However, no significant correlation between serum HBV pgRNA and HBcAb was observed in HBeAg(−) patients (*P* = .089).

### 3.3 Association with HBV DNA load

**Figure 2:**
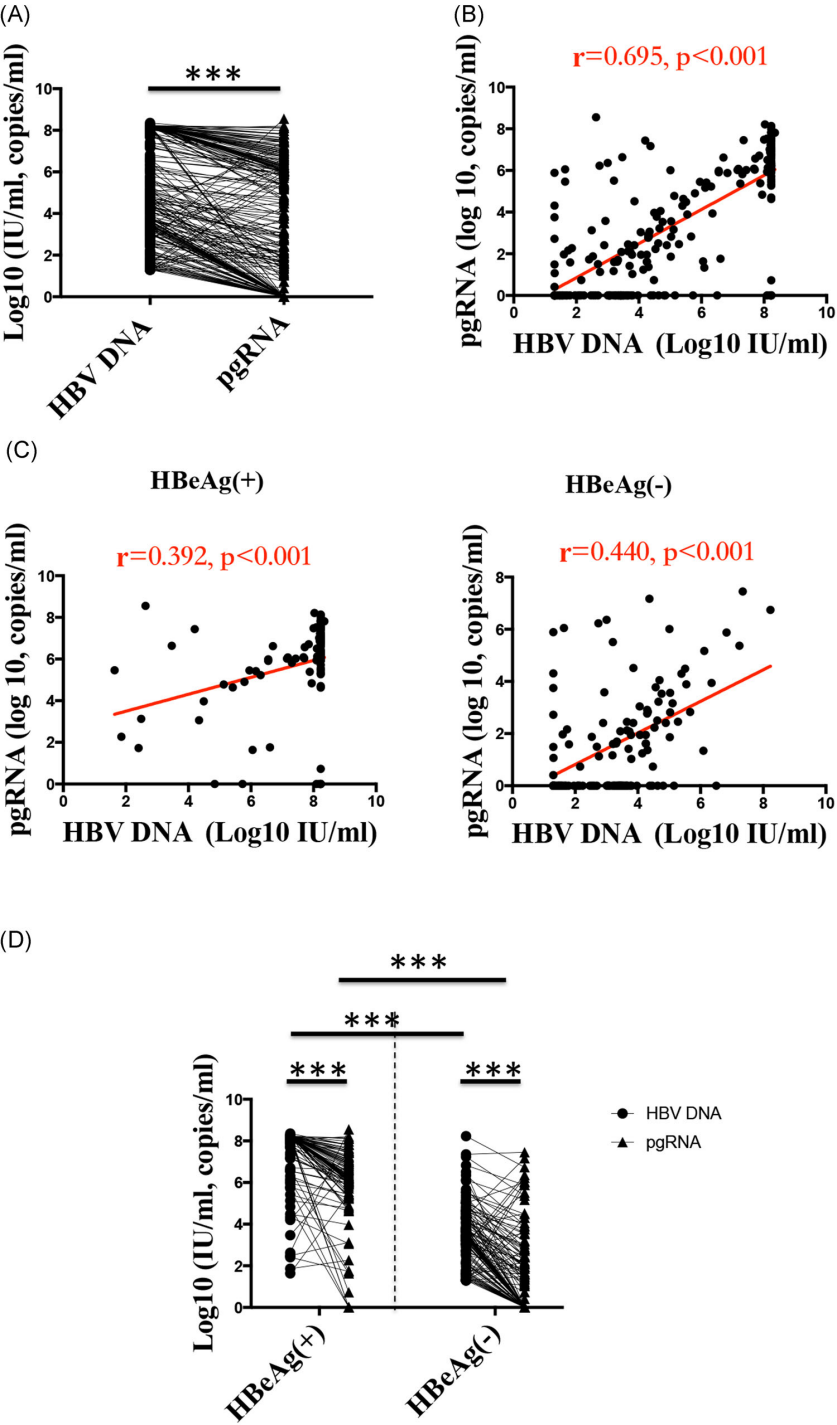
Relationship between serum HBV pgRNA and HBV DNA. A, Serum HBV pgRNA levels were lower than HBV DNA levels in CHB patients. B, C, Correlation between serum HBV pgRNA and HBV DNA in all CHB patients, HBeAg(+) patients and HBeAg(−) patients. D, Serum HBV pgRNA levels were lower than HBV DNA levels in HBeAg(+) and HBeAg(−) CHB patients, and serum HBV pgRNA and HBV DNA levels were all significantly higher in HBeAg(+) patients than in HBeAg(−) patients. CHB, chronic hepatitis B; HBV, hepatitis B virus; pgRNA, pregenomic RNA. *P < .05, **P < .01, ***P < .001

The association between serum levels of HBV pgRNA and HBV DNA was analyzed in a total of 225 subjects. The average serum HBV pgRNA level was 3.13 (0.00, 6.16) log_1_ copies/mL, lower than that of HBV DNA of 4.76 (3.14, 8.23) log_10_ IU/mL in the current CHB cohort (*P* < .001), and in patients with different HBeAg status, using paired analysis (Wilcoxon signed‐rank test) (*P* < .001 and *P* < .001 for patients with HBeAg(+) and HBeAg(−), respectively, Figures 2A and 2D). Serum HBV pgRNA levels were positively correlated with HBV DNA (*r* = .695; *P* < .001) (Figure 2B). When patients were stratified and analyzed by HBeAg status, the Spearman correlation coefficients of serum HBV pgRNA with HBV DNA for HBeAg(+) and HBeAg(−) patients were *r* = .392 (*P* < .001) and *r* = .440 (*P* < .001), respectively (Figure 2C).

As mentioned above, serum HBV pgRNA levels were significantly higher in HBeAg(+) patients than in HBeAg(−) patients (Figure 1D). Similarly, the serum HBV DNA levels in HBeAg(+) patients were higher than those in HBeAg(−) patients (6.19 [5.40, 7.66] vs 1.17 [0.00, 4.78] log_10_ IU/mL; *P* < .001) (Figure 2D). Therefore, compared with HBeAg(−) patients, HBeAg(+) patients had higher levels of both HBV DNA and serum HBV pgRNA, suggesting that the distribution of serum HBV pgRNA levels was similar to that of HBV DNA.

### 3.4 Association with liver inflammation

**Figure 3:**
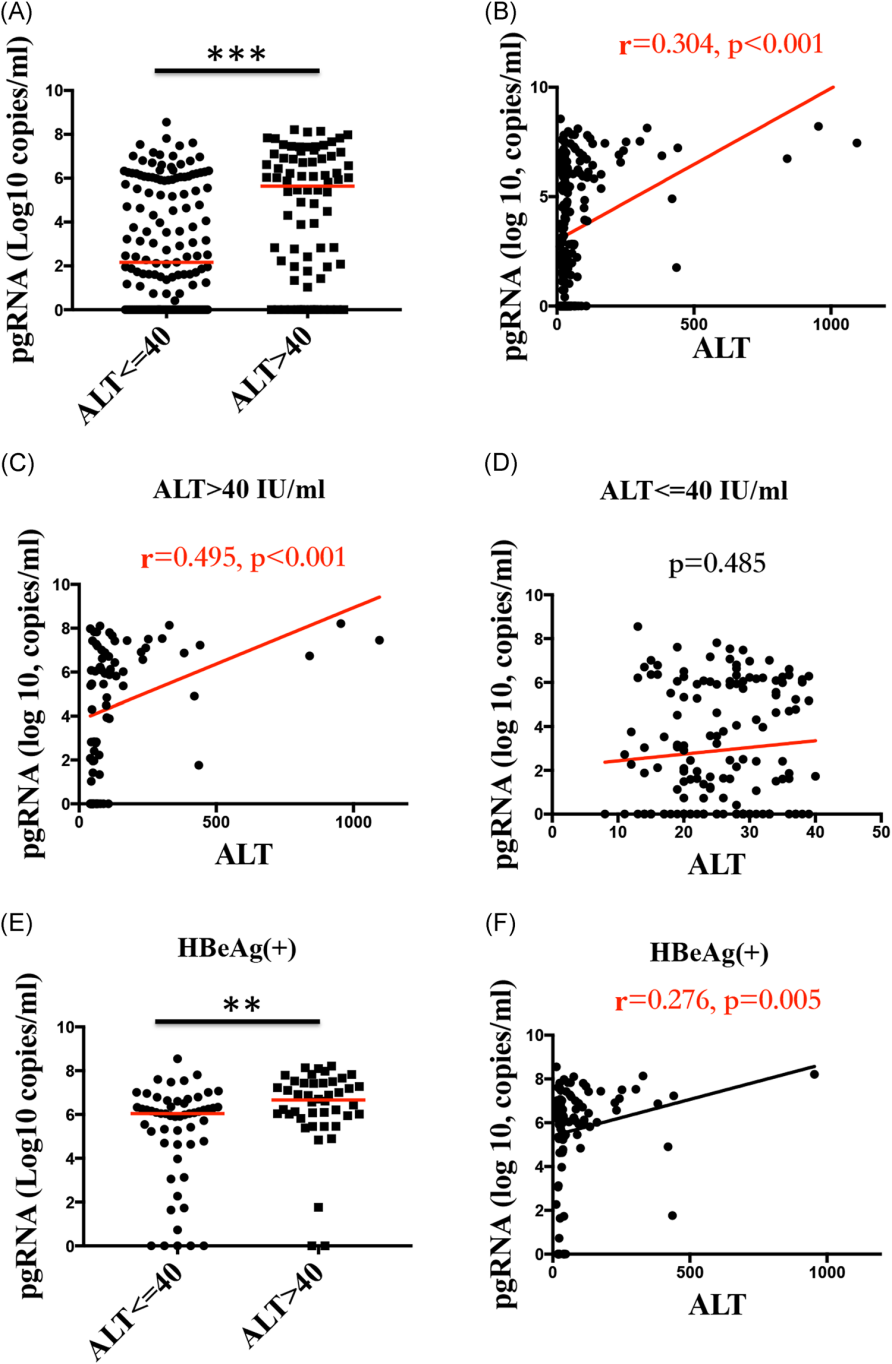
Relationship between serum HBV pgRNA and liver inflammation. A,E, Serum HBV pgRNA levels were higher in patients with elevated ALT (>40 IU/mL) than in patients with normal ALT (≤40 IU/mL) among all patients and among HBeAg(+) CHB patients. B‐D,F, The correlations between serum HBV pgRNA and ALT levels in all CHB patients, patients with elevated ALT, patients with normal ALT and HBeAg(+). CHB, chronic hepatitis B; HBV, hepatitis B virus; pgRNA, pregenomic RNA. *P < .05, **P < .01, ***P < .001

To explore the relationship between serum HBV pgRNA and liver inflammation, serum HBV pgRNA levels were compared between patients with normal ALT ( < = 40 IU/ml) and elevated ALT ( > 40 IU/ml) levels. Compared with patients with normal ALT, patients with elevated ALT had significantly higher serum HBV pgRNA levels [5.64 (1.96, 6.98) vs 2.16 (0.00, 5.93), Log_10_ copies/ml, *P* < 0.001)] (Figure 3a). Moreover, significant correlations were found between the levels of serum HBV pgRNA and ALT in all patients (r = 0.304, *P* < 0.001) (Figure 3b) and in patients with elevated ALT (r = 0.495, *P* < 0.001) (Figure 3c), while the correlation was not significant in patients with normal ALT (*P* = 0.485) (Figure 3d).

To eliminate the effect of virus replication when studying the relationship between serum HBV pgRNA and liver inflammation, serum HBV pgRNA levels were compared between HBeAg(+) patients with normal and elevated ALT levels, whose HBV DNA loads were matched and comparable (*P* = .228). Compared with patients with normal ALT, patients with elevated ALT also had significantly higher serum HBV pgRNA levels (6.66 [5.96, 7.44] vs 6.05 [4.66, 6.58] log_10_ copies/mL; *P* = .002)] (Figure 3E). A significant correlation was found between the serum HBV pgRNA and ALT levels in HBeAg(+) patients (*r* = .276; *P* = .005) (Figure 3F). Because the HBV DNA levels were not comparable in HBeAg(−) patients with normal and elevated ALT levels (*P* = .006), the above analysis was not performed in these patients.

### 3.5 Regression analysis of virological and clinical factors associated with serum HBV PgRNA levels

Univariate linear regression analysis showed that the factors associated with serum HBV pgRNA levels were HBV DNA, qHBsAg, HBcAb level, HBeAg status, genotype C, presence of any C, PreC, or BCP mutations either alone or in combination and ALT levels. By multivariate linear regression, the factors associated with lower serum HBV pgRNA levels were HBV genotype O (mixed genotypes) referencing genotype B (genotype O: *B* = −1.403; *P* = .013), presence of a combination of any C, and PreC or BCP mutation (*B* = −0.755; *P* = .040). Higher ALT (*B* = 0.003; *P* < .001) and HBeAg(+) CHB (*B* = 1.805; *P* = .001) were associated with higher serum HBV pgRNA levels (Table 2). The linear model could explain 56.4% of the serum HBV pgRNA level variance (*r* ^2^ = .564).

**Table 2:** Univariate and multivariate linear regression analysis of factors associated with serum pgRNA

	Univariate		*P* value	Multivariate		Adjusted *P* value
	*B*	95% CI		Adjusted *B*	95% CI	
HBV DNA	0.795	0.693, 0.897	***.000***	0.201	−0.126, 0.527	.227
qHBsAg	1.627	1.319, 1.934	***.000***	−0.228	−0.836, 0.380	.459
HBcAb						
<0.009	Reference			Reference		
≥0.009	−1.056	−1.837, −0.276	***.008***	−0.359	−1.018, 0.301	.284
HBeAg						
Neg	Reference			Reference		
Pos	4.018	3.474, 4.561	***.000***	1.805	0.822, 2.787	***.001***
Genotype						
B	Reference			Reference		
C	0.334	−0.580, 1.247	.472	0.281	−0.416, 0.977	.427
O	−0.939	−2.518, 0.639	.242	−1.403	−2.510, −0.296	***.013***
Mutation						
Wild‐type	Reference			Reference		
C	−1.110	−1.968, −0.252	***.012***	−0.023	−1.641, 1.595	.977
PreC	−1.972	−0.284, −1.104	***.000***	−0.205	−1.147, 0.737	.667
BCP	−1.372	−2.309, −0.435	***.004***	−0.328	−1.217, 0.562	.467
Combined mutation	−1.960	−2.843, −1.076	***.000***	−0.755	−1.476, −0.035	***.040***
ALT	0.007	0.004, 0.010	***.000***	0.003	0.001, 0.005	***.000***

HBV genotype O: besides B or C, including genotype D, B + D, B + C, or C + D.

Abbreviations: BCP, basal core promoter; CI, confidence interval; HBV, hepatitis B virus; pgRNA, pregenomic RNA.

### 3.6 Association with serum cytokines and Th1/Th2 immunity

Because the host immune response plays a major role in CHB pathogenesis, we explored 25 serum inflammatory cytokine profiles associated with serum HBV pgRNA levels by univariate and multivariate linear regression. Analyzed with univariate linear regression, the cytokines associated with serum HBV pgRNA levels were sCD30, IL‐7, IL‐12P40, IP‐10, and MIG‐CXCL9. As determined by multivariate linear regression, sCD30 was positively associated with serum HBV pgRNA levels (*B* = 0.002; *P* = .003), while IL‐12P70 was negatively associated with serum HBV pgRNA levels (*B* = −0.051; *P* = .017) (Table 3).

**Table 3:** Univariate and multivariate linear regression analysis of serum cytokines associated with serum pgRNA

	Univariate		*P* value	Multivariate		Adjusted *P* value
	*B*	95% CI		Adjusted *B*	95% CI	
sCD30	0.002	0.001, 0.003	***.000***	0.002	0.001, 0.003	***.003***
IFN‐γ	0.004	−0.037, 0.045	.847	−0.055	−0.158, 0.049	.457
TNF‐α	0.075	−0.109, 0.260	.419	0.071	−0.468, 0.610	.360
IL‐1β	0.028	−0.017, 0.073	.220	−0.016	−0.165, 0.133	.831
IL‐2	0.010	−0.045, 0.064	.731	0.053	−0.146, 0.253	.596
IL‐4	0.006	−0.014, 0.027	.559	−0.018	−0.080, 0.044	.793
IL‐6	0.001	−0.004, 0.007	.572	0.003	−0.004, 0.011	.550
IL‐7	0.006	0.002, 0.118	***.042***	0.037	−0.047, 0.121	.385
IL‐8	0.001	−0.005, 0.004	.918	0.006	−0.085, 0.096	.904
IL‐12P40	0.046	0.008, 0.084	***.019***	0.045	0.000, 0.092	.055
IL‐12P70	−0.009	−0.047, 0.029	.640	−0.051	−0.092, −0.009	***.017***
IL‐15	0.006	−0.005, 0.016	.295	0.005	−0.01, 0.021	.494
IL‐17α	0.032	−0.189, 0.254	.289	0.004	−2.105, 2.114	.997
IL‐17C	0.264	−0.040, 0.568	.088	0.048	−0.292, 0.388	.779
IL‐22	0.326	−0.001, 0.003	.326	0.001	−0.004, 0.006	.654
IL‐23	0.001	0.001, 0.001	.820	0.001	0.001, 0.001	.556
IL‐28α	0.009	−0.013, 0.030	.420	0.015	−0.005, 0.035	.151
IL‐29	0.001	0.001, 0.001	.616	0.000	−0.004, 0.004	.942
IP‐10	0.006	0.001, 0.011	***.014***	−0.006	−0.014, 0.002	.171
I‐TAC	0.001	0.000, 0.004	.216	−0.002	−0.005, 0.001	.148
GP130	0.001	0.001, 0.001	.714	0.001	0.001, 0.001	.844
MDC‐CCL22	0.001	−0.004, 0.004	.912	−0.001	−0.005, 0.002	.517
MIG‐CXCL9	0.008	0.001, 0.015	***.018***	0.002	−0.022, 0.026	.150
MIP‐1α‐CCL	0.043	−0.027, 0.113	.225	0.024	−0.040, 0.089	.982
MIP‐3α‐CCL2	0.002	0.000, 0.005	.129	0.002	−0.005, 0.009	.587

Abbreviations: CI, confidence interval; IFN‐γ, interferon‐γ; IL, interleukin; pgRNA, pregenomic RNA; TNF‐α, tumor necrosis factor‐α.

**Figure 4:**
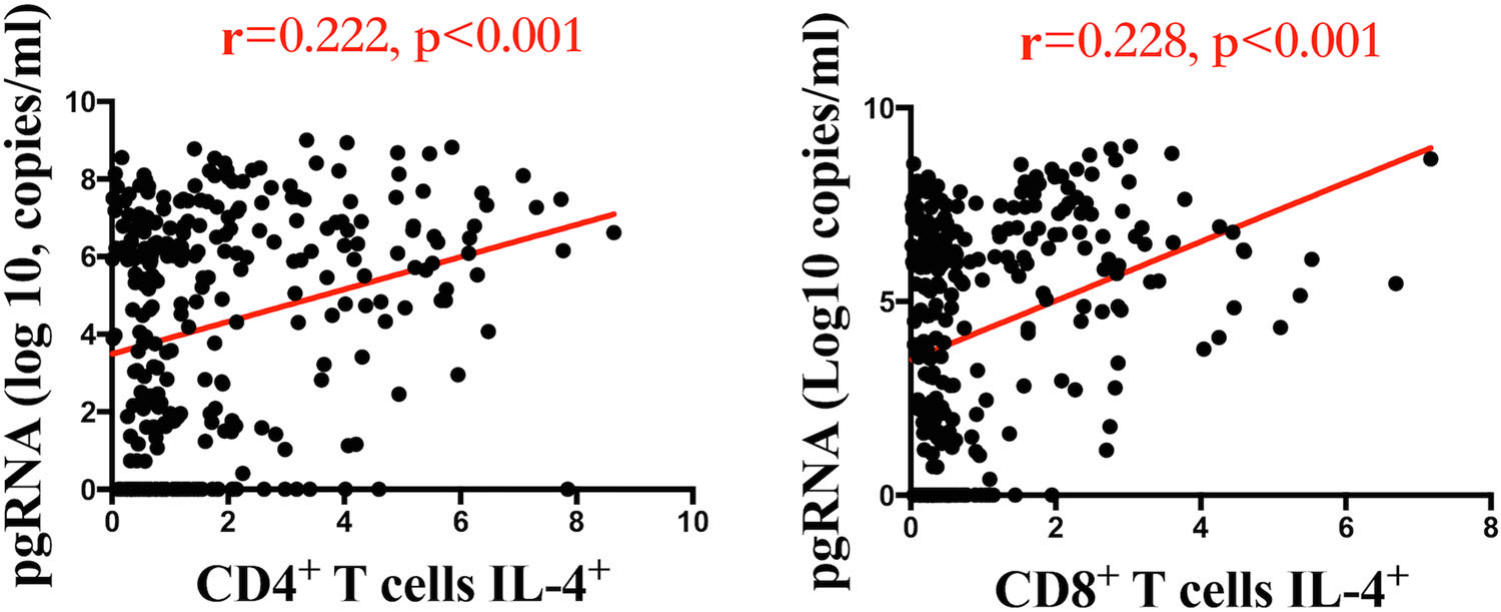
Correlation between serum HBV pgRNA and CD4^+^ T cells producing IL‐4^+^ and CD8^+^ T cells producing IL‐4^+^. HBV, hepatitis B virus; IL‐4, interleukin‐4; pgRNA, pregenomic RNA

CD30 is expressed in activated T cells producing T helper 2 (Th2) cytokines and released as a soluble molecule (sCD30). To better characterize the immunoregulatory role of pgRNA in CHB patients, its association with Th2 cells was further analyzed. A significant positive correlation was found between serum pgRNA levels and CD4^+^ T cells producing IL‐4, which are Th2 cells (*r* = .222; *P* < .001). A positive correlation between serum HBV pgRNA levels and CD8^+^ T cells producing IL‐4 was also found (*r* = .228; *P* < .001) (Figure 4).

IL‐12 is a proinflammatory cytokine that promotes the differentiation of Th1 cells, and Th1 cells enhance the function of cytotoxic T lymphocytes (CTLs). Since serum HBV pgRNA was found to be associated with IL‐12P70, the association of serum HBV pgRNA with Th1 cells, including CD4^+^ and CD8^+^ T cells producing IL‐2, IFN‐γ, and TNF‐α, was evaluated further by univariate and multivariate linear regression. According to multivariate linear regression analysis, serum HBV pgRNA levels showed no association with Th1 cells (*B* = −0.087; *P* = .402), while CD8^+^ T cells producing IFN‐γ, which are CTLs, were negatively associated with serum HBV pgRNA (*B* = −0.052; *P* < .001).

## 4 DISCUSSION

This study elucidated a comprehensive and detailed relationship between serum HBV pgRNA levels and a panel of HBV antigens and antibodies as well as liver inflammation, serum cytokine profiles and Th cell immunity in a cohort of treatment‐naïve CHB patients. Serum HBV pgRNA levels were positively associated with HBV antigens (qHBsAg and HBeAg) and were also correlated with antibodies (HBsAb, HBeAb, and HBcAb). In addition, serum HBV pgRNA levels were positively associated with liver inflammation, Th2, and the Th2 cell‐related cytokine sCD30, but negatively associated with IL‐12P70 and CTLs.

Other researchers have found that serum HBV pgRNA levels are positively correlated with HBV DNA and HBsAg titers in CHB individuals,^21^, ^26^ and our study supports these findings. However, Huang et al^35^ found different situations in HBeAg(+) and HBeAg(−) individuals. In HBeAg(+) patients, the positive correlation between serum HBV pgRNA and HBsAg remained, while serum HBV pgRNA had no correlation with HBsAg in HBeAg(−) individuals. We initially speculated that the lack of correlation in HBeAg(−) individuals was possibly due to an inadequate sample size in a previous study (n = 22). However, a similar result was observed in our study with a larger number of HBeAg(−) patient samples (n = 125), supporting that the disappearance of the correlation might be due to other reasons, such as the host immune response, HBV variation, or cccDNA epigenetic modulation.

Monitoring the intrahepatic cccDNA level is clinically significant for evaluating the antiviral therapy efficacy and predicting the therapy endpoint, which currently relies on liver biopsy. Several obstacles prevent the widespread use of liver biopsies, such as liver invasion, potential complications, and inadequate specimen size.^36^, ^37^ Because serum HBV pgRNA was thought to be a biomarker for the persistence of active intrahepatic cccDNA, we explored the relationship between serum HBV pgRNA and HBV DNA in this study. Similar to intrahepatic cccDNA,^30^, ^31^, ^35^ a positive correlation was also found between serum HBV pgRNA and HBV DNA by both correlation and linear regression analyses, indicating that serum HBV pgRNA might be a suitable marker for persistent HBV infection.

Early studies indicated that serum pgRNA was found in exosomes.^38^, ^39^ Chou et al^40^ found that HBV‐infected cells noncytolytically secrete nonenveloped (“naked”) capsids that contained all HBV DNA replicative intermediates, including pgRNA. Wang et al^19^ reported that serum HBV pgRNA levels did not relate to exosome secretion. They further confirmed that serum HBV pgRNA originated from encapsidated virus‐like particles. However, the mechanism of the release of pgRNA into serum from infected hepatocytes remains unclear. Wang et al^17^ found that serum HBV RNA levels were obviously correlated with necroinflammation histopathological scores, and one of their mechanistic explanations was that HBV RNA produced in hepatocytes could be released into circulation in proportion to the intensity of liver cell necroinflammation. In a study by van Campenhout et al,^33^ HBV RNA levels were significantly higher in patients with higher ALT levels than in those with lower ALT levels. In our results, serum HBV pgRNA levels were also positively correlated with ALT in patients with comparable HBV DNA levels, supporting other researchers’ opinions that serum HBV pgRNA particles could be released from injured hepatocytes.^17^ Furthermore, we found that serum HBV pgRNA was associated with Th1/Th2 immunity, which plays an important role in liver inflammation, potentially contributing to the association of serum HBV pgRNA with ALT.

HBV DNA fragments can integrate into the human genome and produce serum HBsAg, suggesting that serum HBsAg is not an optimal viral marker of intrahepatic cccDNA replication activity in CHB patients.^41^, ^42^, ^43^, ^44^, ^45^, ^46^ Serum HBV pgRNA is a new and important marker for intrahepatic HBV replication that could predict early NUC efficacy and prognosis of CHB patients and be used to monitor HBV resistance during NUC therapy.^19^, ^20^, ^21^, ^26^, ^32^, ^35^ Indeed, we herein found that serum HBV pgRNA was significantly correlated with most of the commonly used HBV replication markers, such as HBV DNA, HBsAg, and HBeAg status, which supports that serum HBV pgRNA might be an optional marker to reflect intrahepatic HBV replication activity. However, one should rule out the effect of ALT and HBV mutations when assessing HBV replication with this potential indicator because both of these mutations were also well correlated with serum HBV pgRNA.

CD30, a membrane receptor of the tumor necrosis factor superfamily, is expressed on activated Th2 cells and released into sera as a soluble molecule (sCD30). The positive association between serum HBV pgRNA and sCD30 in CHB patients reflected the relationship between serum HBV pgRNA and the activation of Th2 cells. A significantly positive correlation was also found between serum HBV pgRNA levels and CD4^+^ T cells producing IL‐4, which are Th2 cells, and further supported the relationship of serum HBV pgRNA with Th2 cells. Th2‐like responses play an important role in humoral immunity responses, and our results further showed that serum HBV pgRNA was associated with HBV antigens and antibodies. HBeAg(−) patients who were positive for both HBsAg and HBsAb had higher serum levels of HBV pgRNA than HBeAg(−) patients with negative HBsAb, and patients with higher HBcAb values had a significantly lower serum HBV pgRNA level. Serum HBV pgRNA levels were different among patients with different HBeAg and HBeAb statuses. All these results indicated that serum HBV pgRNA might have a relationship with factors related to humoral immunity in CHB patients, such as HBV antigen seroconversion and antibody production. However, in this study, serum HBV pgRNA was negatively correlated with the cellular immunity of CTLs and with IL‐12P70, which promotes the differentiation of Th1 cells. Many studies have confirmed that the HBV DNA load and virus antigens, such as HBsAg and HBeAg, are related to the innate and adaptive immunity of CHB patients, however, the relationship between serum HBV pgRNA and host immunity is not known. The results of our study provide new insight into the relationship of serum HBV pgRNA with host immunity.

In conclusion, we found that serum HBV pgRNA is related to not only virus antigens and antibodies but also to Th cell immunity in treatment‐naïve CHB patients. The results demonstrated that serum HBV pgRNA was positively correlated with Th2 immunity but negatively correlated with Th1 immunity, indicating that serum HBV pgRNA might have a relationship with HBV antigen conversion and CTL immunodeficiency in CHB patients.

## CONFLICT OF INTERESTS

The authors declare that there are no conflict of interests.

## Supporting information

Supporting information

Supporting information
