# Supplementary figures and images for: Serum HBV pregenomic RNA is correlated with Th1/Th2 immunity in treatment‐naïve chronic hepatitis B patients
Source: J Med Virol. 2019 Nov 21;92(3):317–28. doi: 10.1002/jmv.25612 (PMC7004183; doi:10.1002/jmv.25612)

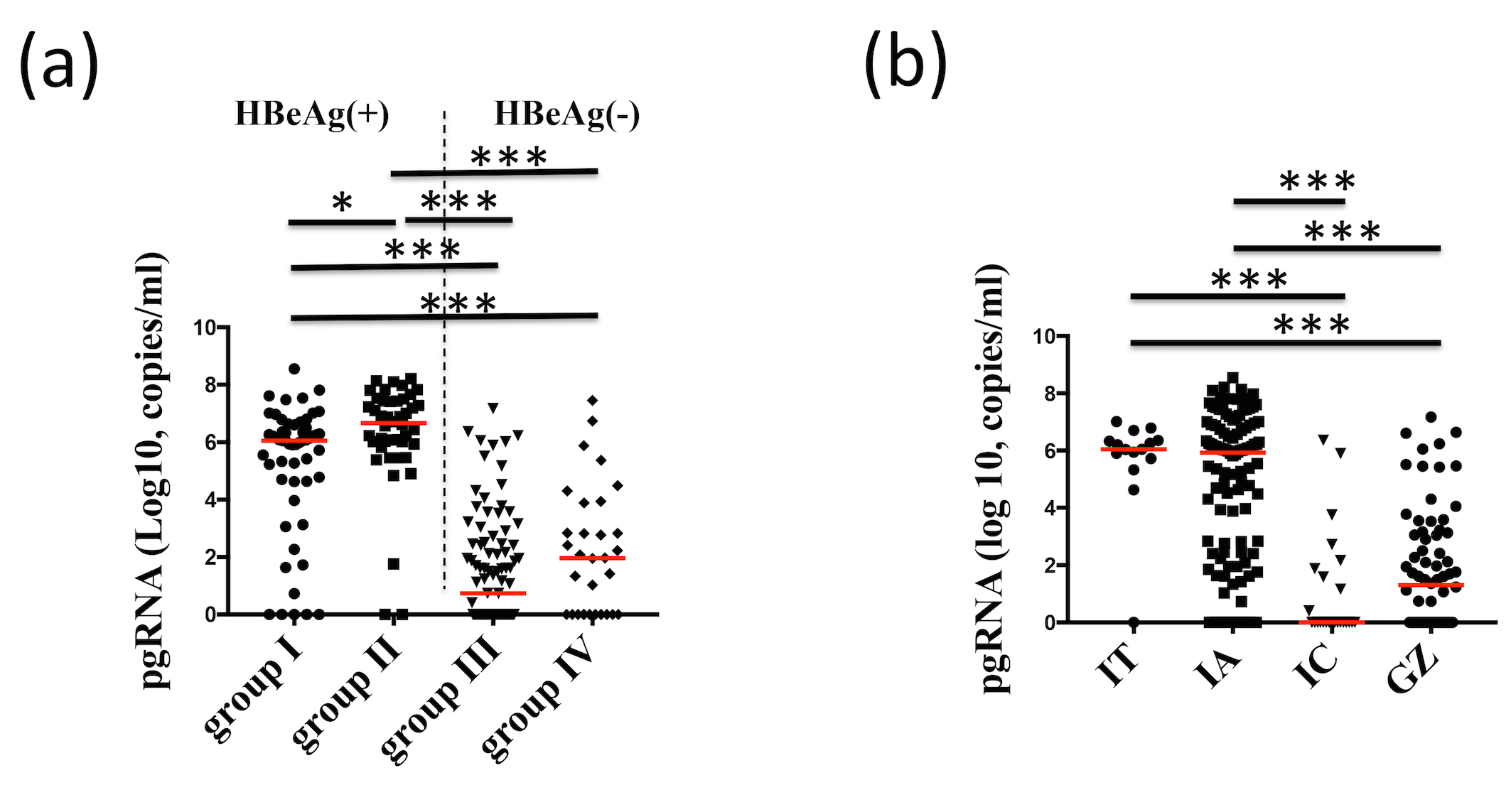

Supplement: Supplementary file 1 — Supporting information [file JMV-92-317-s001.tif]
