# Supplementary material for: Serum HBV pregenomic RNA is correlated with Th1/Th2 immunity in treatment‐naïve chronic hepatitis B patients
Source: J Med Virol. 2019 Nov 21;92(3):317–28. doi: 10.1002/jmv.25612 (PMC7004183; doi:10.1002/jmv.25612)
Supplement: Supplementary file 2 — Supporting information [file JMV-92-317-s002.docx]

**Supplementary figure 1. Distribution of serum HBV pgRNA levels in the current cross-sectional study.** (a) Serum HBV pgRNA levels were higher in group II patients [HBeAg(+) and ALT>40 U/L] than in the other three groups, and were also significantly higher in group I patients [HBeAg(+) and ALT40≤ U/L] than in group III [HbeAg(-) and ALT40≤ U/L] and group IV [HbeAg(-) and ALT>40 U/L] patients. (b) Distribution of serum HBV pgRNA levels among the patients in immune tolerant (IT), immune active (IA), inactive carriers (IC) and gray zone (GZ) phases. The levels in both of the former two phases were higher than those in the latter two phases. *p<0.05, **p<0.01, ***p<0.001.
